# Increase in *Vibrio parahaemolyticus* Infections Associated with Consumption of Atlantic Coast Shellfish — 2013

**Published:** 2014-04-18

**Authors:** Anna E. Newton, Nancy Garrett, Steven G. Stroika, Jessica L. Halpin, Maryann Turnsek, Rajal K. Mody

**Affiliations:** 1 Division of Foodborne, Waterborne, and Environmental Diseases, National Center for Emerging Infectious and Zoonotic Disease, CDC

*Vibrio parahaemolyticus* (*Vp*) is found naturally in coastal saltwater. In the United States, *Vp* causes an estimated 35,000 domestically acquired foodborne infections annually (1), of which most are attributable to consumption of raw or undercooked shellfish. Illness typically consists of mild to moderate gastroenteritis, although severe infection can occur. Demographic, clinical, and exposure information (including traceback information on implicated seafood) for all laboratory-confirmed illnesses are reported by state health departments to CDC through the Cholera and Other *Vibrio* Surveillance system. *Vp* isolates are distinguished by serotyping (>90 serotypes have been described) and by pulsed-field gel electrophoresis (PFGE).

*Vp* serotypes O4:K12 and O4:K(unknown) comprise the Pacific Northwest (PNW) strain and, within the United States, had not been associated with shellfish outside the Pacific Northwest before 2012. During May–July 2012, *Vp* of the PNW strain associated with shellfish from Oyster Bay Harbor in New York caused an outbreak of 28 illnesses in nine states. Simultaneously, *Vp* of the PNW strain caused an outbreak of illnesses on a cruise ship docked on the Atlantic Coast of Spain; illness was associated with cooked seafood cooled with ice made from untreated local seawater. All *Vp* isolates from ill persons in the U.S. and Spanish outbreaks that were further subtyped were indistinguishable by PFGE (2).

In 2013, this same indistinguishable strain was traced from shellfish consumed by ill persons to a larger area of the U.S. Atlantic Coast, causing illness in 104 persons from 13 states during May–September (Figure). The median age of patients was 51 years (range = 22–85 years); 62% were male. Six (6%) patients were hospitalized; none died. Multiple outbreaks appeared to be occurring, accounting for many of these illnesses. Illness was associated with consumption of raw shellfish and seafood traceback was reported for 59 (57%) illnesses. Of these illnesses, 51 (86%) involved seafood that could be definitively traced to a single harvest area. The implicated harvest areas were located in Connecticut (20 illnesses), Massachusetts (15), New York (10), Virginia (four), Maine (one), and Washington (one). The remaining eight illnesses with traceback information involved seafood that could not be definitively traced to a single harvest area (locations reported included harvest areas of the Atlantic Coast of the United States and Canada). In response to the illnesses, four Atlantic Coast states closed implicated harvest areas; two issued shellfish recalls (3). The number of foodborne *Vp* cases in the United States traced to Atlantic Coast shellfish was threefold greater in 2012 and 2013 compared with the annual average number reported during 2007–2011.

This PNW strain is possibly becoming endemic in an expanding area of the Atlantic Ocean. The mechanisms for this introduction are not known. During the 2014 *Vibrio* season, beginning in the spring, clinicians, health departments, and fisheries departments should be prepared for the possibility of shellfish-associated diarrheal illness caused by this strain again. Appropriate actions, such as quick closure of implicated harvest areas, will help prevent additional illnesses. The Interstate Shellfish Sanitation Conference maintains a list of shellfish harvest area closures and recalls.* Clinicians seeking an etiology of diarrhea in a patient who has recently consumed raw or undercooked shellfish should notify the microbiology laboratory that *Vp* is suspected; the use of special culture media (thiosulfate citrate bile salts sucrose) facilitates identification of *Vibrio* species. Consumers can reduce their risk for *Vp* infection by avoiding eating raw or undercooked shellfish, especially oysters and clams.†

## Figures and Tables

**FIGURE:**
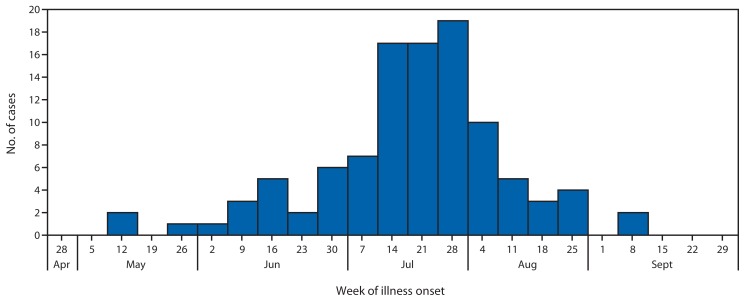
*Vibrio parahaemolyticus* illnesses (N = 104) associated with consumption of shellfish from Atlantic Coast harvest areas, by week of onset — United States, 2013

## Footnotes

* Available at http://www.issc.org/closuresreopenings.aspx.

† Additional information available at http://www.cdc.gov/vibrio/investigations/vibriop-09-13/advice-consumers.html.
